# Lung Cancer with Isolated Pleural Dissemination as a Potential ctDNA Non-Shedding Tumor Type

**DOI:** 10.3390/cancers17152525

**Published:** 2025-07-30

**Authors:** Huizhao Hong, Yingqian Zhang, Mengmeng Song, Xuan Gao, Wenfang Tang, Hongji Li, Shirong Cui, Song Dong, Yilong Wu, Wenzhao Zhong, Jiatao Zhang

**Affiliations:** 1 Guangdong Lung Cancer Institute, Guangdong Provincial People’s Hospital (Guangdong Academy of Medical Sciences), Southern Medical University, Guangzhou 510000, China; hzhong2015@163.com (H.H.); twf2012@qq.com (W.T.); hjli16@fudan.edu.cn (H.L.); dsong@aliyun.com (S.D.); syylwu@live.cn (Y.W.); 2 Geneplus-Beijing Institute, Beijing 102206, China; zhangyingqian7@outlook.com (Y.Z.); songmm@geneplus.org.cn (M.S.); 3 State Key Laboratory of Microbial Resources, Institute of Microbiology, Chinese Academy of Sciences, Beijing 100101, China; gaoxuan@geneplus.org.cn; 4 GenePlus-Shenzhen Clinical Laboratory, Shenzhen 518122, China; 5 Department of Cardiothoracic Surgery, Zhongshan City People’s Hospital, Zhongshan 528403, China; 6 Operational Research, Department of Industrial Engineering and Operations Research, Fu Foundation School of Engineering and Applied Science, Columbia University, New York, NY 10027, USA; sc4914@columbia.edu

**Keywords:** non-small cell lung cancer, pleural dissemination, ctDNA, non-shedding

## Abstract

**Objectives:** Circulating tumor DNA (ctDNA) has emerged as a reliable prognostic biomarker in both early- and late-stage non-small cell lung cancer (NSCLC) patients. However, its role in NSCLC with pleural dissemination (M1a), a subset of disease with indolent biology, remains to be elucidated. **Methods:** We collected 41 M1a patients with serial ctDNA and CEA monitoring. Progression-free survival (PFS) was assessed between patients with different levels of ctDNA and CEA. An independent cohort of 61 M1a patients was included for validation. **Results:** At the diagnostic landmark, the detection rates for ctDNA and CEA were 22% and 55%, respectively. Among patients who experienced disease progression with pleural metastases, only ten had detectable ctDNA in longitudinal timepoints, resulting in a sensitivity of 50%. Moreover, there was no significant difference in PFS between patients with longitudinally detectable and undetectable ctDNA (HR: 0.86, 95% CI 0.33–2.23, *p* = 0.76). In contrast, patients with a decreasing CEA trend within 3 months after diagnosis were associated with an improved PFS (HR: 0.22; 95% CI, 0.03–1.48, *p* = 0.004). This finding is confirmed in an independent M1a patient cohort. **Conclusions:** Together, our findings suggest that M1a NSCLC with isolated pleural dissemination may represent a “non-shedding” tumor type, where ctDNA shows limited diagnostic and prognostic value. Monitoring early changes in CEA could be a more cost-effective predictor of disease progression.

## Simple Summary

Recent studies have highlighted the predictive and prognostic value of circulating tumor DNA (ctDNA) in non-small cell lung cancer (NSCLC). However, its clinical utility may vary across different tumors based on their biology, which remains largely unknown. We aimed to investigate the role of ctDNA in NSCLC with pleural dissemination (M1a), a subset of advanced disease characterized by an indolent biology. We found that M1a NSCLC demonstrates a ctDNA non-shedding phenotype, with a low detection rate and limited prognostic value. In contrast, the early increase in carcinoembryonic antigen (CEA) levels was associated with poor prognosis in this patient population. These results suggest that despite advances in high-throughput methods, CEA retains an important clinical role. For patients with M1a NSCLC, longitudinal CEA monitoring should be implemented as part of routine clinical management.

## 1. Introduction

Lung cancer is the leading cause of cancer-related death worldwide, accounting for 350 deaths per day [1]. Stage IV lung cancer represents the most advanced disease [2]. However, a subset of patients with isolated pleural seeding (M1a) has shown better survival outcomes [2,3,4] and may benefit from surgical resection of the primary tumor [4,5]. Previously, we identified a group of M1a patients without lymph node metastasis and with low genomic instability, which may be associated with an indolent biology. These patients may benefit from surgical resection followed by a wait-and-see strategy. In contrast, some M1a patients with high tumor burden and instable genomic characteristics may experience a rapid disease progression within a few months, even with continuous treatment [3]. Therefore, the discovery of biomarkers to stratify patients and predict disease progression is crucial for optimizing surveillance and management for these patients.

Circulating tumor DNA (ctDNA) is derived from tumor cells and evaluated using deep next-generation sequencing [6]. Recent studies have highlighted the prognostic value of ctDNA, where a positive ctDNA signal post-treatment is associated with an elevated risk of progression across both early and advanced stages of cancer [7,8,9]. Conversely, our previous cohort study showed that longitudinal undetectable ctDNA after radical surgery may signify a cure status [9]. However, in clinical practice, we noticed that ctDNA was insensitive to disease progression in some patients, including those with M1a disease. Moreover, the majority of ctDNA studies involve patients at various stages, which may obscure its performance in certain populations. How ctDNA performs in the specific type of M1a patients is unclear. Additionally, the non-shedding characteristic of a tumor, defined as the presence of a gross tumor without the release of ctDNA into the blood, is considered to reflect indolent biological behavior [9,10,11]. How the discrepant biology of M1a disease impact the prognostic value remains to be fully understood.

Serum carcinoembryonic antigen (CEA) is a traditional tumor marker, which was widely used as a supplementary monitoring biomarker in routine clinical practice. A vast majority of published studies have elucidated its prognostic and predictive role in various cancer, including colorectal cancer [12,13,14], gastric carcinoma [15,16], gynecologic cancer [17,18], hepatocellular carcinoma [19], and lung cancer [20,21,22,23,24]. Despite its established role, the nonspecific nature of CEA has limited its prognostic utility. Notably, in clinical practice, we observed sensitivity to CEA changes in certain patients, especially those insensitive to ctDNA. Nowadays, the specific prognostic role of serum CEA in M1a patients remains uncertain.

Herein, we retrospectively collected and analyzed serial ctDNA and CEA samples from patients with M1a disease and explored the potential for ctDNA-based and CEA-based disease monitoring in this patient population.

## 2. Materials and Methods

### 2.1. Ethics Statement

The experimental protocols, sample collection, and clinical data acquisition carried out in this study were approved by the Research Ethics Committee of Guangdong Provincial People’s Hospital, Guangdong Academy of Medical Sciences (KY-Q-2021-202-01). We collected written informed consent from all participants.

### 2.2. Study Population and Clinical Data Collection

**Figure 1:**
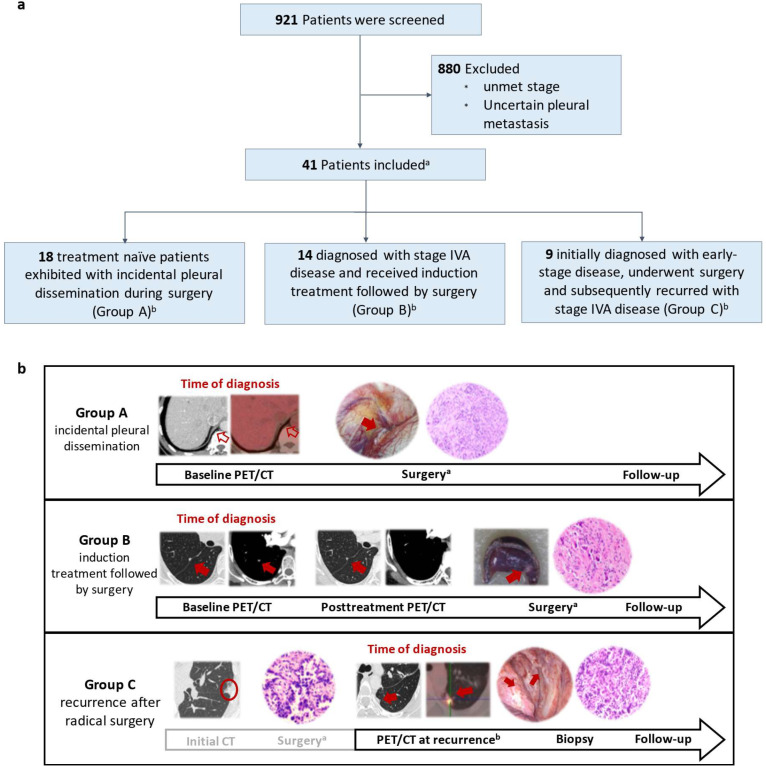
Summary of patient enrollment. (**a**) Study flowchart. This diagram illustrates the patient enrollment process. ^a^ All enrolled patients were diagnosed with stage IVA disease (AJCC 8th TNM) with either pleural dissemination and were followed up with ctDNA-MRD and radiological assessments. ^b^ Patient follow-up began from the time of diagnosis with M1a disease and continued until the event of extrathoracic progression or death occurred. Specifically, Group A and Group B patients were followed from the time of initial diagnosis, whereas Group C patients were followed from the time of disease recurrence. (**b**) Treatment courses of 3 types of patients. Group A included treatment naïve patients with incidental pleural dissemination identified during surgery. Group B patients were initially diagnosed with stage IVA disease and underwent induction treatment followed by resection of the primary tumor. Group C patients were those who had undergone prior radical resection of the primary tumor and later developed pleural recurrence without distant metastasis. Red arrows indicate the site of representative pleural dissemination lesions. In Group A, no detectable lesions were observed on radiological examination; the arrow points to the site of incidental pleural dissemination identified during surgery. The red circle marks the primary lung tumor. ^a^. Surgical resection of primary tumor. ^b^. PET/CT showing pleural recurrence.

From July 2018 to September 2024, we retrospectively screened 921 patients with non-small cell lung cancer (NSCLC) who were treated at Guangdong Provincial People’s Hospital. Eligible patients were diagnosed with stage IVA disease according to the AJCC 8th edition TNM staging system. Patients who had suspicious pleural dissemination lesions were confirmed by pathologic examination and/or positron emission computed tomography (PET/CT), regardless of lymph node status, were considered eligible. Patients with distant or undetermined pleural metastases during surgery were excluded. A total of 41 patients were included and allocated into three groups according to their treatment course: incidental pleural seeding during surgery (group A); induction treatment followed by surgery (group B), and recurrent pleural dissemination after resection (group C) (Figure 1).

For the eligible patients, we collected their detailed clinical information from medical documents, including age, sex, smoking history, tumor size, stage (AJCC 8th edition), lymph node status, F-FDG uptake on PET/CT, histological type, and treatment as well. Patients were followed up every 3–6 months using computed tomography (CT) or PET/CT and blood tests. Prognostic data were collected from outpatient clinics and telephone interviews. The last follow-up date was 28 September 2024.

To validate the prognostic significance of CEA, another cohort of patients diagnosed with stage IVA (AJCC 8th edition TNM staging system) with pleural dissemination and with serial CEA monitoring was obtained from the Guangdong provincial people hospital database.

### 2.3. Sample Collection

Peripheral blood samples were collected before and after the time of M1a diagnosis, and matched tissue samples obtained from primary tumors during surgery were used for mutation profiling. Peripheral blood (16 mL) was collected in two 10 mL streak tubes. Landmark ctDNA is defined as the blood samples collected before patient treatment. Longitudinal ctDNA refers to blood samples collected at least 3 days after the diagnosis and every 3–6 months thereafter.

### 2.4. Library Construction and Next Generation Sequencing

DNA from tumor tissue samples and plasma samples were sheared into 200~250 bp fragments with the Covaris S2 ultrasonicator (Covaris, Inc., Woburn, MA, USA), libraries were constructed using the NEBNext Ultra DNA Library Prep Kit for Illumina (NEB, Ipswich, MA, USA), germline gnomic DNA from peripheral blood lymphocytes was used as the reference genome to identify single nucleotide polymorphisms (SNPs) in tumor DNA and cfDNA for each patient. Next, DNA libraries were hybridized to a previously reported custom-designed 1021-panel (Integrated DNA Technologies, Inc., Coralville, IA, USA) (Supplementary Table S1). Then, indexed libraries were sequenced on the DNBSEQ-T7RS sequencer (MGI Tech, Shenzhen, China) using the 100-bp paired-end method.

### 2.5. Raw Data Processing and Tumor Somatic Variant Calling

The raw sequenced data were mapped to the reference human genome (GRCh37) using BWA-0.7.17 (r1188), with default parameters after removing adaptor and low-quality reads. Duplicated reads were identified and removed using MarkDuplicates in GATK. Local realignment around SNVs and indels (insertion and deletion) as well as quality control assessments were executed using GATK. Tumor somatic SNVs and indels were then profiled using Mutect2, CNVs were detected using AllelicCNV, and the structural variations were analyzed using lumpy.

### 2.6. ctDNA-MRD Detection

After data processing and variant calling followed by annotation completion, variants were filtered according to the following criteria: (i) variants present in matched genomic DNA were removed; (ii) the single-nucleotide polymorphisms at >1% population allele frequency in ExAc or 1000 Genomes Project were filtered; and (iii) the variant positional depth was at least >200×.

Furthermore, cfDNA variants which met the following criteria were considered to be true somatic mutations: (i) for hotspot mutations, ≥4 high-quality support reads, or for non-hotspots, at least ≥8 support reads; and (ii) clonal hematopoiesis were filtered through deep sequencing of paired white blood. A plasma sample with at least one variant detected was defined as ctDNA-positive.

Overall, the sequencing coverage and quality statistics for each sample including the tumor tissue and plasma are summarized in Supplementary Table S2.

### 2.7. CEA Test

The serum CEA concentration was measured retrospectively using the chemiluminescence immunoassay (Beckman Coulter Ref. 33200, Indianapolis, IN, USA). Peripheral blood (4 mL) was collected in a 4 mL ethylenediaminetetraacetic acid tube before and after M1a diagnosis. The definitions of landmark and longitudinal CEA correspond to diagnostic landmark and longitudinal ctDNA above. Early CEA response is defined as the changes of CEA levels within three months following the diagnosis of M1a disease.

### 2.8. Statistical Analysis

The time of progression was evaluated using CT or PET/CT. Progression-free survival (PFS) was calculated from the time of M1a diagnosis to disease progression or patient’s death (Figure 1). Kaplan–Meier analysis was used to assess survival outcomes. Univariate and multivariate analyses using the Cox proportional hazard model explored the clinicopathologic factors associated with survival outcomes. *p* < 0.05 in a two-tailed test was interpreted as a significant difference. We used R version 4.2.1 (R Foundation for Statistical Computing, Vienna, Austria), Prism version 9.00 (GraphPad Software, La Jolla, CA, USA), and Adobe Illustrator 2021 (Adobe Systems Incorporated, San Jose, CA, USA) for statistical analysis, graphic generation, and modification.

## 3. Results

### 3.1. Subsection

#### 3.1.1. Baseline Characteristics

A total of 41 M1a patients were analyzed, including 18 Group A (44%), 14 Group B (34%), and 9 Group C (22%) patients. (Figure 1) The median age of this cohort was 60 years (range: 32–79), with the majority being male (51%), never-smokers (56%) and diagnosed as adenocarcinoma (95%). The median primary tumor size was 2.7 cm (range: 0.7–4.4 cm), and approximately half of the patients were N0 (23/29, 56%). All patients had an ECOG performance status of 0–1 and underwent surgical resection of the primary tumor. Detailed clinicopathological characteristics of M1a patients are presented in Table 1.

We identified 341 mutations in 40 of the 41 tumor specimens, with a median of 2 mutations per sample (1–26 mutations). Detected mutations were classified into 283 single-nucleotide mutations, 40 deletions, 8 insertions, and 10 insertions/deletions. Of the 341 mutations (mapped to 163 genes), 206 were identified as tumor drivers (73 genes). EGFR (70%) and TP53 (50%) were the two most frequently mutated genes (Supplementary Figure S1).

#### 3.1.2. M1a Lung Cancer Is a Potential Non-Shedding Tumor

A total of 216 blood samples were tested for ctDNA. A total of 184 ctDNA mutations were detected, and 80% of the detected mutations matched those in tumor samples. Moreover, 140 of 184 mutations were identified as tumor drivers (mapped to 35 of 61 genes). *EGFR* (60%) and *TP53* (48%) were the most prevalent mutated genes (Supplementary Figure S1).

**Figure 2:**
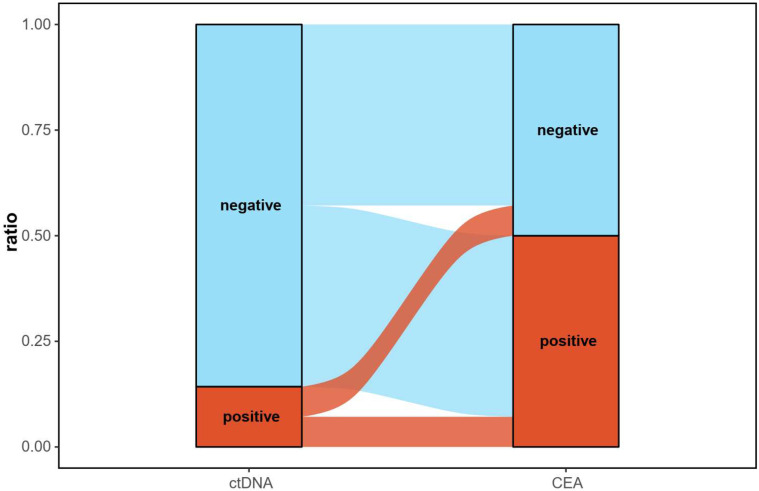
Diagnostic landmark of ctDNA and CEA. The left column shows the proportion of patients with detectable versus undetectable ctDNA. The right column depicts the distribution of CEA levels, categorized as positive (CEA ≥ 5 ng/mL) or negative (CEA < 5 ng/mL), at the diagnostic landmark. Among patients with paired ctDNA and CEA data at the diagnostic landmark, 79% of patients had undetectable ctDNA and 50% had positive CEA. Of patients with undetectable ctDNA, 55% were CEA-positive, whereas among patients with detectable ctDNA, 67% were CEA-negative.

To evaluate the diagnostic efficacy of ctDNA and the shedding ability of tumor cells, we analyzed the ctDNA results taken prior to patient treatment. Of the 41 M1a patients, 18 had ctDNA testing before treatment. The positive diagnostic rate of ctDNA was 22%, with Group B patients demonstrating the highest detection rate (Group A: 8%; Group B: 67%; Group C: 33%). The overall positive diagnostic rate of ctDNA is substantially lower than 55% of CEA. Interestingly, when we analyzed the paired CEA results at the diagnostic landmark, 55% (6/11) of patients with initially undetectable ctDNA signals turned into a positive CEA result (Figure 2). These findings suggest that in cases of pleural dissemination, ctDNA might not be consistently shed into the bloodstream. In contrast, serum CEA appears to offer superior diagnostic value in these scenarios, highlighting a discrepancy in biomarker shedding and detection between ctDNA and CEA in certain clinical context.

#### 3.1.3. ctDNA Is Ineffective in Predicting Progression in Patients with M1a Disease

The median follow-up period was 35.5 months (range: 4.1–78.0 months). The median PFS was 20.4 months. Disease progression occurred in 27 patients, including 18 intrathoracic and 9 extrathoracic events. The brain (seven out of nine, 78%) was the most common site of extrathoracic progression. The others were abdominal lymph nodes and bone metastases.

**Figure 3:**
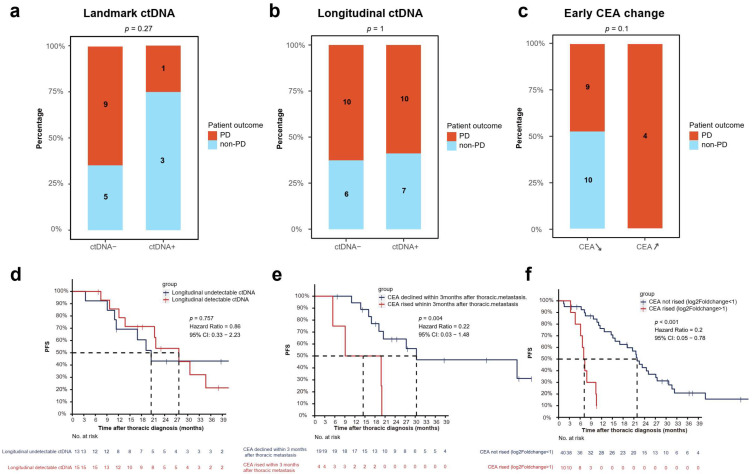
Survival outcomes by ctDNA and CEA results. (**a**) Detection rate of landmark ctDNA in patients who experienced disease progression and those who maintained stable disease. (**b**) Detection rate of longitudinal ctDNA in patients who experienced disease progression and those who maintained stable disease. (**c**) Analysis of early changes in CEA levels within the first three months after diagnosis in patients with progressive disease versus stable disease. (**d**) Kaplan–Meier analysis of progression-free survival (PFS) in patients with detectable versus undetectable longitudinal ctDNA. (**e**) Kaplan–Meier analysis showing PFS in patients where CEA levels either increased or decreased within three months of diagnosis. (**f**) Validation of the Kaplan–Meier PFS analysis based on early CEA changes in an independent patient cohort. CEA level with a log2Fold change > 1 compared to the baseline data is defined as CEA with a rising trend. A rise in CEA levels was defined as a log2Fold change > 1 when compared to baseline values, indicating an increasing trend in CEA.

We then investigated whether ctDNA could predict disease progression. Initially, we assessed the prognostic significance of ctDNA at the diagnostic landmark. Among the ten patients who experienced disease progression, only one had detectable landmark ctDNA, yielding a sensitivity of 10% (Figure 3a). Conversely, three out of eight patients with a detectable ctDNA signal at this diagnostic juncture remained with stable disease throughout the study period. To further evaluate ctDNA dynamics, we monitored ctDNA levels longitudinally. ctDNA demonstrated limited sensitivity of 50%, with only ten patients showed detectable ctDNA prior to disease progression (Figure 3b). Survival analysis indicated no difference in PFS between patients with longitudinal undetectable and detectable ctDNA (HR: 0.86, 95% CI 0.33–2.23, *p* = 0.76) (Figure 3d). These findings suggest that ctDNA has limited prognostic utility in patients with M1a disease.

#### 3.1.4. Early Serum CEA Response May Be a Cost-Effective Biomarker for M1a Patients

Since ctDNA is an ineffective prognostic biomarker for M1a disease, the identification of other biomarkers is necessary for the prognosis of these patients. Certain M1a patients presented with substantially elevated CEA levels followed by disease progression within a few months after diagnosis (Supplementary Figure S2). We proposed that CEA levels could potentially be applied for disease monitoring in such patients. We collected 352 serial CEA test results from our patient population. A total of 39 out of 41 patients had at least two CEA results. The median level of CEA was 4.8 ng/mL (range: 0.4–297.8 ng/mL).

The fluctuation in serum CEA levels within three months following the diagnosis of pleural dissemination offers a potential indicator of treatment response. Among the thirteen patients who subsequently experienced disease progression, four (31%) showed an increase in CEA levels within this period. Notably, all ten patients whose CEA level declined within the first 3 months after diagnosis maintained a stable disease (Figure 3c). The positive predictive value (PPV) of an elevated CEA trend within three months after diagnosis was found to be 100%, indicating a high reliability in predicting disease progression. Patients with increased CEA within this timeframe had a higher progression risk than those whose CEA levels decreased during this period (HR: 0.22; 95% CI, 0.03–1.48, *p* = 0.004) (Figure 3e).

To validate the robustness of early serum CEA response as a prognostic marker, we included an independent cohort of 61 patients diagnosed with stage IVA disease and pleural dissemination. This cohort comprised patients with a median age of 55 years (range: 23–75 years), with predominantly male (67%) and never-smokers (73%). All patients had adenocarcinoma, with 61% presenting with N0 stage disease at baseline (61%). Serial CEA monitoring was performed in these patients. Patients were categorized into three groups according to the documented treatment courses (Supplementary Table S3). Median PFS was significantly shorter with an upward CEA trend, defined as log2 foldchange > 1 within three months after the diagnosis versus those with decreased trend. (HR: 0.2; 95% CI, 0.05–0.78, *p* < 0.001) (Figure 3f). This result reinforced that early changes in serum CEA levels could be a more reliable prognostic biomarker than ctDNA in this specific patient population.

## 4. Discussion

Patients with M1a require a long treatment period and careful follow-up. Moreover, some of these patients experience diverse outcomes, with an OS longer than 5 years without any treatment, whereas others who receive continuous treatment show rapid cancer progression [3]. Therefore, discovery of a robust biomarker enables an accurate prediction of disease progression and treatment guidance is essential.

In this study, we examined the diagnostic and prognostic value of ctDNA for patients with solely pleural dissemination. We found that M1a lung cancer may represent a non-shedding tumor type. Moreover, ctDNA was insufficient for the identification of patients at a high risk of progression. CEA is a well-known tumor marker for lung cancer. When we evaluated the efficacy of CEA levels as a prognostic tool, we observed an elevated trend of serum CEA levels within the first 3 months after diagnosis, which may indicate a high risk of progression.

Moreover, M1a patients presented with low ctDNA landmark detection rates, which were substantially lower than those previously reported in patients with advanced-stage lung cancer [25]. We considered several possible reasons for this. Firstly, a number of factors, such as indolent biology and restricted biological activity, may have contributed to the low ctDNA release by M1a lung cancer. Prior studies suggested that ctDNA shedding may reflect tumor biology and tumor burden, especially in cancers of a biologically aggressive phenotype, exhibiting rapid proliferation and a large tumor mass [9,10]. M1a patients have a five-year OS rate of 10%, which is generally higher than that of stage IV patients [2]. Second, in contrast to distant metastases, which mostly exhibit major tumor vasculature, pleural metastatic nodules are located in a serous membrane supplied by small branches of intercostal or bronchial vessels. Tumor cells are therefore less likely to release ctDNA into the circulatory system. Furthermore, most patients had undetectable ctDNA signals with the increasing number and volume of pleural nodules, which highlights the “non-shedding” nature of pleural nodules. In contrast, M1a with malignant pleural effusion was associated with vascular epithelial growth factor and inflammatory factor production, which promoted angiogenesis and increased vascular permeability, consequently generating exudative pleural effusion with malignant tumor cells [26,27,28]. In addition, anti-angiogenic therapy has demonstrated promising efficacy in such patients [29,30]. Therefore, patients with M1a and malignant pleural effusion may have a higher probability of ctDNA detection. We assumed that M1a patients, specifically those with dry pleural dissemination, have “non-shedding” tumors.

ctDNA can be utilized for the detection of tumors, the prediction of their progression, and for guiding the clinical decision-making process [7,8,9]. However, the potential of ctDNA detection in non-shedding tumors has remained largely elusive. Our study showed that neither landmark nor longitudinal ctDNA could identify M1a patients with a high risk of progression, regardless of whether they had intrathoracic or extrathoracic progression. Among patients who experienced disease progression, most exhibited progression within the chest wall and presented with an increased density of pleural nodules, enlarged pleural nodules, or pulmonary spreading. For those with extrathoracic progression, seven out of nine patients developed brain metastasis [9,31]. The unsatisfactory prognostic value of ctDNA in patients with M1a warrants the exploration of additional biomarkers. Moreover, further research is required to determine the role of ctDNA in other non-shedding tumors.

CEA is a 200 kDa glycoprotein derived from the endodermal epithelium during the embryonic phase and was first described as a tumor-related marker by Freedman and Gold in 1965 [32]. CEA may play a role in tumorigenesis in light of its value as a tumor marker [33]. In lung cancer patients, elevated CEA levels were associated with a shorter PFS and OS [20,21,23,24], while low CEA levels indicated a favorable outcome [21,22]. However, a retrospective study of 341 patients with stage I lung cancer determined that preoperative CEA had no impact on prognosis [34]. Meanwhile, advanced-stage patients with high baseline serum CEA but low baseline cytokeratin 19 fragment (CYFRA 21-1) had a longer OS [35]. In the present study, we discovered that, instead of preoperative or postoperative CEA, an elevated CEA trend could predict M1a disease progression. Moreover, the elevated CEA level trend within 3 months after diagnosis had the highest prognostic value, similar to a previous study [24]. This result suggests that the serum CEA test is more cost-effective in stratifying patients with M1a disease into high- and low-risk groups during the early follow-up period.

The present study had certain limitations. First, owing to the low incidence of M1a disease, a relatively small number of patients were analyzed. Therefore, subgroup analysis was limited. To improve the reliability of our results, we excluded patients with undetermined pleural seeding even though suspected pleural nodules were observed during surgery. Patients diagnosed with stage IVA without pleural dissemination were also excluded from the analysis. However, patient selection bias was inevitable due to the single-center and real-world nature of our study.

## 5. Conclusions

In conclusion, we identified that M1a with dry pleural dissemination may represent a “non-shedding” tumor. In accordance, ctDNA detection was not effective in M1a disease monitoring. Serum CEA may be a more cost-effective biomarker for predicting disease progression.

## Figures and Tables

**Table 1:** Clinical characteristics of the enrolled patients.

	M1a Patients *n* = 41 (%)
PS score	
0~1	41 (100%)
2	0 (0)
Age (median)	60 (32–79)
Gender	
Male	21 (51%)
Female	20 (49%)
Smoking history	
Never smoker	23 (56%)
Ever/Current smoker	18 (44%)
Histology	
Adenocarcinoma	39 (95%)
Mucinous adenocarcinoma	1 (2%)
Adenosquamous carcinoma	1 (2%)
Tumor size (cm) (median)	2.7 (0.7, 4.4)
Lymph node status	
N0	23 (56%)
N1	5 (12%)
N2	12 (29%)
N3	1 (2%)
Population	
Group A	18 (44%)
Group B	14 (34%)
Group C	9 (22%)
First-line treatment	
Targeted therapy	23 (56%)
Immunotherapy	7 (17%)
Chemotherapy	2 (5%)
Others	9 (22%)
Survival	
Intrathoracic progression	18 (44%)
Extrathoracic progression	9 (22%)
Survival without progression	14 (34%)

PS, performance status.

## Data Availability

The datasets used and/or analyzed during the current study are available from the corresponding authors on reasonable request.

## Supplementary Materials

The following supporting information can be downloaded at: https://www.mdpi.com/article/10.3390/cancers17152525/s1, Figure S1. Heatmap showing the baseline characteristics and mutations in each timepoints. Figure S2. Dynamic monitoring of ctDNA and CEA in a 60-year-old female diagnosed with stage IVA with pleural dissemination. This patient received induction erlotinib for 3 months, followed by surgical resection of the primary tumor. Pathological examination of the adenocarcinoma and pleural dissemination was performed during surgery. After surgery, the patient received first-line treatment with erlotinib for 25 months, followed by second-line therapy with osimertinib. The time to progression for this patient was 12 months. After that, the number and size of pleural nodules increased gradually. Intrapulmonary dissemination arose in both lungs. At 24th months after surgery, pleural effusion was identified during a chest computed tomography (CT) scan. This patient had an undetectable ctDNA result at the landmark and longitudinal timepoint until 24 months after surgery. In addition, CEA testing revealed an elevated CEA trend 3 months after diagnostic compared to the landmark CEA result, with the serum CEA level gradually increasing from 6 to 24 months after surgery. Supplementary Table S1. 1021-Targeted panel for tumor tissues. Supplementary Table S2. Summary of sequencing coverage and quality statistics. Supplementary Table S3. Summary of independent cohort

## Abbreviations

The following abbreviations are used in this manuscript:

CEA	Carcinoembryonic antigen
CT	Computed tomography
ctDNA	Circulating tumor DNA
NSCLC	Non-small cell lung cancer
OS	Overall survival
PET/CT	Positron emission computed tomography
PFS	Progression-free survival
