# Supplementary material for: Lung Cancer with Isolated Pleural Dissemination as a Potential ctDNA Non-Shedding Tumor Type
Source: Cancers (Basel). 2025 Jul 30;17(15):2525. doi: 10.3390/cancers17152525 (PMC12346199; doi:10.3390/cancers17152525)
Supplement: Supplementary file 1 [file cancers-17-02525-s001.zip › Supplementary Figures.pdf]

Supplementary Figures

Supplementary Figure S1

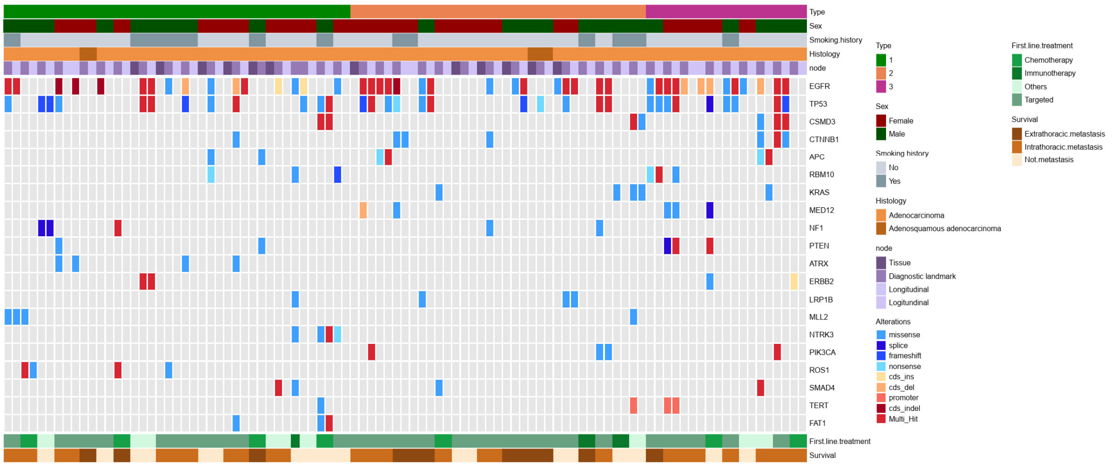

Supplementary Figure S1. Heatmap showing the baseline characteristics and mutations in each timepoints.

Supplementary Figure S2

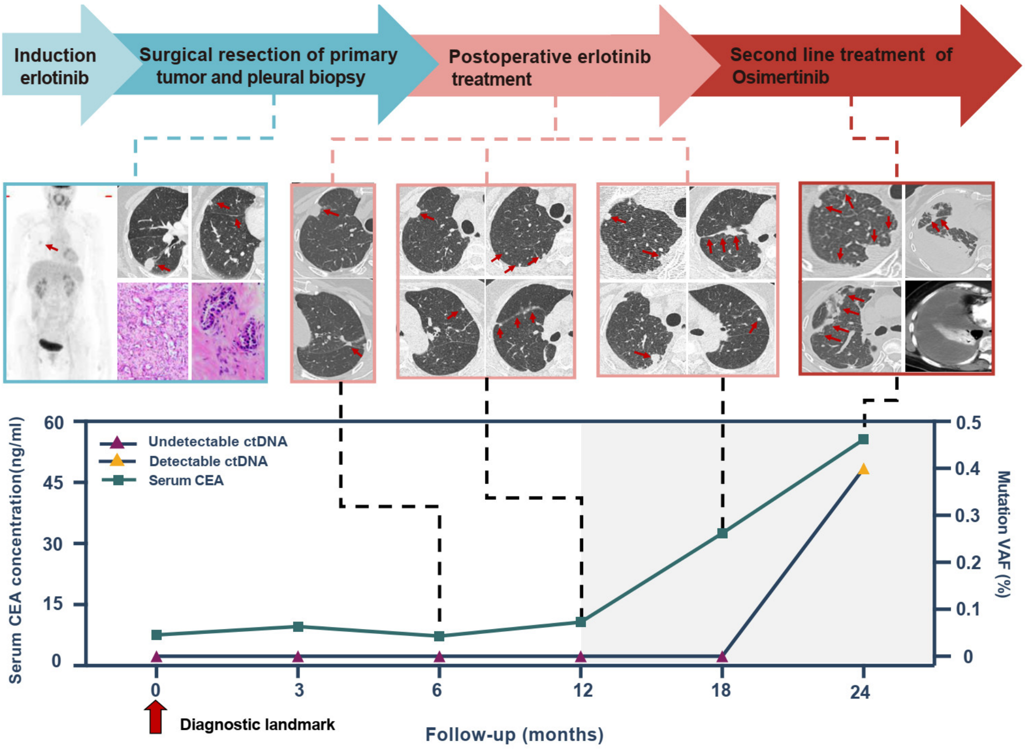

Supplementary Figure S2. Dynamic monitoring of ctDNA and CEA in a 60-year-old female diagnosed with stage IVA with pleural dissemination. This patient received induction erlotinib for 3 months, followed by surgical resection of the

primary tumor. Pathological examination of the adenocarcinoma and pleural dissemination was performed during surgery. After surgery, the patient received first-line treatment with erlotinib for 25 months, followed by second-line therapy with osimertinib. The time to progression for this patient was 12 months. After that, the number and size of pleural nodules increased gradually. Intrapulmonary dissemination arose in both lungs. At 24th months after surgery, pleural effusion was identified during a chest computed tomography (CT) scan. This patient had an undetectable ctDNA result at the landmark and longitudinal timepoint until 24 months after surgery. In addition, CEA testing revealed an elevated CEA trend 3 months after diagnostic compared to the landmark CEA result, with the serum CEA level gradually increasing from 6 to 24 months after surgery.
