# Supplementary material for: Lung Cancer with Isolated Pleural Dissemination as a Potential ctDNA Non-Shedding Tumor Type
Source: Cancers (Basel). 2025 Jul 30;17(15):2525. doi: 10.3390/cancers17152525 (PMC12346199; doi:10.3390/cancers17152525)
Supplement: Supplementary file 1 [file cancers-17-02525-s001.zip › Supplementary Table S3.pdf]

Supplementary Table S3. Summary of independent cohort

	**M1a patients N=61 (%)**
**Age (median)**	55 (23–75)
**Gender**	
Male	41 (67%)
Female	20 (33%)
**Smoking history**	
Never smoker	45 (73%)
Ever/Current smoker	16 (26%)
**Histology**	
Adenocarcinoma	61 100%)
**Tumor size (cm) (median)**	2.8 (1.0, 9.0)
**Lymph node status**	
N0	37 (61%)
N1	6 (10%)
N2	15 (25%)
N3	3 (4%)
**Population**	
Group A	37 (61%)
Group B	3 (5%)
Group C	21 (34%)
**Survival**	
Progression	50 (82%)
Survival without progression	11 (18%)
